# Evaluation of acetic acid treatment of fresh–cut water chestnuts using gray–correlation analysis based on the variation–coefficient weight

**DOI:** 10.3389/fnut.2024.1370611

**Published:** 2024-06-20

**Authors:** Yongqing Zhang, Haiyan Li, Jiangfan Liu, Quanzeng Wei, Juntao Sun, Deguo Wang

**Affiliations:** Key Laboratory of Biomarker–Based Rapid Detection Technology for Food Safety of Henan Province, Xuchang University, Xuchang, China

**Keywords:** acetic acid, water chestnut, fresh-cut fruits and vegetables, food preservation, gray correlation analysis, variation coefficient method

## Abstract

**Introduction:**

The demand for fresh–cut water chestnuts, a convenient and nutritive vegetable, is increasing in market. However, the slicing of water chestnuts can cause mechanical damage to tissue, which results in quality deterioration. We aimed to select the optimal treatment through a comprehensive comparison of the preservation effect of acetic acid, which could prolong the shelf life of fresh–cut water chestnuts and improve their storage quality.

**Methods:**

A comprehensive evaluation was conducted using the gray–correlation method based on the variation–coefficient weight to observe the treatment of 0, 2 and 5% acetic acid. Their effects on color, weight loss rate, and the content of ascorbic acid, total sugar, reducing sugar, soluble protein, and free amino acid were determined.

**Results:**

The color, weight loss rate, and nutritional content of fresh–cut chestnuts varied under different processing and storage times. When stored for more than 4 days, the b^*^ value, and the content of total sugar and soluble protein in CK were higher than those in 2% or 5% acetic acid, but the weight loss rate, and the content of ascorbic acid and free amino acid in CK were less than those in acetic acid treatments. Considering various indicators, it was difficult to determine which treatment to choose for fresh–cut water chestnut preservation. The gray–correlation analysis results indicated that when stored for 8, 12, or 16 days, the gray–correlation degree of 5% acetic acid was the highest, while that of the control was the lowest. It could be directly concluded by the gray–correlation degree that when the storage time exceeded 4 days, acetic acid could be used to improve storage quality, and 5% acetic acid had a better preservation effect than 2%. Fresh–cut water chestnuts can be stored for 4 days without the need for acetic acid treatment.

**Conclusion:**

These findings could provide information and comprehensive evaluation methods for the preservation of fresh–cut fruits and vegetables. The next step is to evaluate the preservation effect of acetic acid by measuring its effects on other indicators of fresh–cut water chestnuts (e.g., flavonoids, and microorganisms), providing ideas for the research of preservatives.

## 1 Introduction

Water chestnut (*Eleocharis tuberosa*) belongs to the Cyperaceae family and is cultivated in China, India, the United States, Vietnam, and South Korea. It contains 68% water and 21.8% carbohydrates (1).Water chestnuts are also rich in vitamins, minerals, and other compounds, which have anticancer, antibacterial, laxative, and diuretic properties (1, 2). However, when eating water chestnuts, the skin should be peeled off. Fresh–cut water chestnuts can be eaten raw as fruits or processed together with other foods to meet the growing demand of consumers. However, the slicing of water chestnuts can cause mechanical damage to tissue, which results in quality deterioration, such as browning and nutrient loss. The edible and commercial values of fresh–cut chestnuts have been severely affected. Therefore, effectively controlling the deterioration of fresh–cut chestnuts to achieve preservation effect and ensure their nutritional content remains a challenge, which hinders the rapid development of the water chestnut industry.

The main preservation methods for fresh–cut water chestnuts include physical, chemical, and biological methods. Physical methods mainly include heat treatment (3), N_2_ (4) and high-pressure carbon dioxide treatment (5), etc., while exogenous salicylic acid (6) and hydrogen peroxide (7) are chemical methods. Chitosan coating (8), rice bran extracts (9) and eugenol (10) are biological methods. Composite preservation method can also be used, such as a combination of ultraviolet irradiation and ethanol (11). However, soaking and coating can easily lead to high residual levels of reagents in fresh–cut water chestnuts, and the effectiveness of air conditioning treatment and heat treatment is not as good as that of preservatives, or specialized equipment is required (12). Water chestnut, widely planted in Hubei, Guangxi, Zhejiang, Hunan, and other places, is one of the important aquatic vegetables in China, with a total area of 50,000 hm^2^, and the annual output of fresh fruit of water chestnut is 600,000–800,000 tons. Developing safe, effective, convenient, and low–cost preservatives will contribute to the development of the fresh–cut water chestnut industry.

Acetic acid is the active ingredient of vinegar, which has the advantages of easy volatility, low residue, and low cost; therefore, acetic acid is considered as an environmentally friendly liquid (13, 14).Anhydrous acetic acid, also known as glacial acetic acid, is a safe food additive approved for use in China (GB 2760–2014). Acetic acid is not only a sour and flavoring agent, but also an antibacterial component in vinegar and an effective preservative to prevent food spoilage (15). The reason why acetic acid prevents fruit and vegetable spoilage may be because it reduces the respiratory intensity of the product and inhibits the growth of surface microorganisms (16–18). Acetic acid could preserve fresh corn (19), maintain the post–harvest quality of tomatoes (20), and also serve as a substitute for sulfur dioxide to prevent Kyoho grapes decay (21). Fresh–cut water chestnuts gradually turn yellow during storage, and the discoloration is caused by non–enzymatic and enzymatic browning (22). According to report, *Geotrichum*, *Streptococcus*, and *Staphylococcus* seem to play a dominant role in causing severe yellowing of fresh–cut water chestnuts (23). Therefore, acetic acid may be able to maintain the freshness of fresh–cut water chestnut, but little is known about its application in the preservation of fresh–cut water chestnut.

In order to provide scientific basis for the application of acetic acid in preservation, it is necessary to measure multiple quality indicators of fresh–cut water chestnuts as much as possible. The large amount of data obtained may cause confusion in understanding the relationship between various indicators or their contribution to the evaluation of the preservation effect of acetic acid on fresh–cut water chestnuts. Therefore, it is necessary to establish a scientific comprehensive evaluation method for the quality of fresh–cut water chestnuts. Gray correlation method is a multivariate statistical analysis method that calculates the absolute value of data differences between sequences (24). The multi–objective optimization of the Q355C steel gas metal arc–welding process (25) and the process parameters for plastic injection molding (26) was performed by the gray–correlation method. The correlation degrees between the frost–penetration depths and some parameters, such as thermal characteristics and average annual temperatures, were also described by gray–correlation analysis (27). The method was also used in safety evaluation of chemical process (28), quality evaluation of Chinese medicinal materials (29), and food processing characteristics (30–33). The present study focused on determining the color, weight loss rate, and some nutritional components of fresh–cut water chestnuts exposed to different concentrations of acetic acid solutions. The experimental data were analyzed and compared. Then, the importance of different concentrations of acetic acid solutions affecting the quality indicators of fresh–cut water chestnuts was classified by using the gray–correlation method based on the variation–coefficient weight. Our findings may provide information and a comprehensive evaluation approach to the preservation of fresh–cut water chestnuts and other fresh–cut fruits and vegetables.

## 2 Materials and methods

### 2.1 Plant materials

Water chestnut was obtained from a commercial market in Xuchang. The fruit was processed in accordance with the method of Peng and Jiang (6), which was selected for uniformity and size, and any bruised or diseased fruit was discarded, washed, peeled, and cut into 5 mm–thick slices using a stainless–steel knife. The prepared slices were randomly divided into three groups. One group that served as the control was soaked in pure water for 5 min, and the other two groups that served as the test groups were soaked in 2 and 5% glacial acetic acid solutions for 5 min, respectively. After dripping water for 10 min, each of the three groups was initially divided into 15 parts, then, respectively, weighed and packaged through heat sealing using PE preservation bags, and finally stored at 4°C in a BCD–133EN Haier refrigerator (Qingdao Haier Co., Ltd., Qingdao, Shandong, China). Sampling and measuring of relevant indicators were performed at 0, 4, 8, 12, and 16 days. Each treatment was repeated three times.

### 2.2 Application of gray–correlation analysis based on the variation–coefficient weight in quality evaluate of fresh–cut water chestnuts

The color, weight loss rate, and content of nutritional components in fresh–cut water chestnuts were measured under different processing and storage times. A large amount of data was collected to conduct a scientific comprehensive evaluation for the quality and acetic acid preservation effect of fresh–cut water chestnut.

#### 2.2.1 Quality determination

1. Color determination. The color of fresh–cut water chestnuts was determined using a NR200 colorimeter (Shenzhen Sanenchi Technology Co., Ltd., Shenzhen, China). L^*^ (Lightness) and b^*^ (yellow), the two color parameters, were used to indicate the color of fresh–cut water chestnuts. The colorimeter was standardized with a white plate before each test.
2. Weight loss rate. The weight loss rate is the difference in sample weight before and after storage. It was calculated using Equation 1.

(1) $$\mathrm{Weight}\;\mathrm{loss}\;\mathrm{rate}\;\left(\%\right)=\frac{x_{0}-x_{i}}{x_{0}}\times 100$$

where $x_{0}$ is the weight of fresh–cut water chestnuts before packaging; $x_{i}$ is the weight of fresh–cut water chestnuts stored for i day; i refers to 0, 4, 8, 12, or 16 days.

1. Ascorbic acid. The total ascorbic acid was measured with a UV–visible photometer (T6 New Century, Beijing Puche General Instruments Co., Ltd., Beijing, China) at 500 nm using the colorimetric method described by GB/T 5009.86–2003 (China).
2. Total sugar. The content of total sugar was detected with a UV–visible photometer (T6 New Century, Beijing Puche General Instruments Co., Ltd., Beijing, China) at 620 nm using the Anthrone colorimetric method (34). Extraction was conducted as follows. One gram of fresh–cut water chestnuts was weighed in a mortar, ground evenly, and transferred to a 150 mL conical flask. The mortar was rinsed with 15 mL of pure water, and the washing liquid was transferred into the Erlenmeyer flask, added with 10 mL of 6 mol/L hydrochloric acid, immediately cooled in ice water after a 30–min boiling water bath, neutralized with 20% sodium hydroxide solution, transferred to a 100 mL volumetric flask at a constant volume, and filtrated. Afterward, 2 mL of the filtrate was sucked into another 100 mL volumetric flask at a constant volume. One milliliter of the diluted extract was pipetted into a stoppered test tube, and 4 mL of Anthrone reagent was added to the test tube. Finally, the absorbance value was measured.
3. Reducing sugar. The reducing sugar was detected with a UV–visible photometer (T6 New Century, Beijing Puche General Instruments Co., Ltd., Beijing, China) at 540 nm using the 3, 5–dinitrosalicylic acid method (35).
4. Soluble protein. The soluble protein was detected with a UV–visible photometer (T6 New Century, Beijing Puche General Instruments Co., Ltd., Beijing, China) at 595 nm using the Coomassie brilliant blue dye method (35).
5. Free amino acid. Free amino acid was detected with a UV–visible photometer (T6 New Century, Beijing Puche General Instruments Co., Ltd., Beijing, China) at 570 nm using the ninhydrin colorimetric method (35).

#### 2.2.2 Variation–coefficient weighting method

Variation–coefficient method is a statistic to measure the variation degree of observed values in data. The standard deviation can be used directly if the unit of measurement is the same as the average. If the unit is different from average, the standard deviation cannot be used to compare the degree of variation, but the ratio of the standard deviation to the average should be used to compare the degree of variation (36). The coefficient of variation calculated by the steps below can avoid the equal division of weight and make the result more reasonable (37).

1. Calculating the coefficient of variation. The coefficient of variation of the indicator was calculated using Equation 2 (36).

(2) $$V_{i}=\frac{\delta_{i}}{X_{i}}$$

where $V_{i}$ is the coefficient of variation of the indicator *Ci*; $\delta_{i}$ is the standard deviation of the indicator *Ci*; $X_{i}$ is the mean value of the indicator *Ci*; *i* = 1, 2, …, *n*, and *n* is the number of indicators (*n* = 1, 2, …, 8).

1. Calculating the objective weight. The objective weight of the indicator was calculated using Equation 3 (36).

(3) $$W_{i}=\frac{V_{i}}{\sum_{i=1}^{n}V_{i}}$$

where *Wi* is the object weight of the indicator *Ci*; *Vi* is the variation coefficient of the indicator *Ci*, and *n* is the number of indicators (*n* = 1, 2, …, 8).

#### 2.2.3 Gray–correlation analyses

Gray system theory is mainly applied to uncertain systems where certain information is known and unknown. It uses relatively small datasets and does not require observable values to strictly follow certain statistical laws (24, 38). Gray correlation analysis based on gray system theory is a multivariate statistical analysis used to evaluate the correlation between different sequences in design (27). In removing the dimensional differences of each indicator, the impact of different concentrations of acetic acid on the quality of fresh–cut water chestnuts was evaluated through gray–correlation analysis based on color, weight loss rate, ascorbic acid, total sugar, reducing sugar, soluble protein, and free amino acid. In the following, analysis procedure for gray correlation degree is described.

1. Normalizing raw data. To eliminate the impact of different dimensions and orders of magnitude on quality assessment, it is necessary to initialize quality indicators (39). That is, raw data is needed to be normalized. In order to standardize the raw data, the ideal concentration was selected as the reference sequence (X0), and other concentrations (CK, 2, and 5% acetic acid) were used as the comparison sequence (Xj). In addition to the weight loss rate, the minimum value of b^*^ and the maximum value of other quality indicators in the comparison sequence were the corresponding values for ideal processing. The weight loss rate of the ideal processing was the smallest value among those values of weight loss rate in the comparison sequence except for 0. The raw data were preprocessed and converted into dimensionless data using Equation 4 (40).

(4) $$C_{i}^{\prime }\left(k\right)=\frac{C_{i}\left(k\right)}{c_{0}\left(k\right)\;}$$

where *i* is the number of samples (*i* = 0, 1, 2, …, 15), and *k* is the number of indicators (*k* = 1, 2, …, 8).

1. Determining the absolute difference between comparison and reference sequence. The absolute difference was calculated using Equation 5 (38).

(5) $$\Delta_{i}\left(k\right)=|C_{0}^{'}\left(k\right)-C_{0}^{'}\left(k\right)|$$

The minimum absolute difference was measured using Equation 6.


(6) $$\Delta \min=\mathrm{minmin}|C_{0}^{\prime }\left(k\right)-C_{i}^{\prime }\left(k\right)|$$

The maximum absolute difference was calculated using Equation 7.


(7) $$\Delta \max=\mathrm{maxmax}|C_{0}^{\prime }\left(k\right)-C_{i}^{\prime }\left(k\right)|$$

where *i* is the number of samples (*i* = 0, 1, 2, …, 15), and *k* is the number of indicators (*k* = 1, 2, …, 8).

1. Calculating the gray–correlation coefficient. The gray–correlation coefficient is considered as the correlation between the ideal and actual standardized value. This coefficient can be calculated using Equation 8 (38).

(8) $$\zeta_{i}\left(k\right)=\frac{\Delta \min+\rho \Delta \max}{\Delta_{i}\left(k\right)+\rho \Delta \max}$$

where *i* is the number of samples (*i* = 0, 1, 2, …, 15); *k* is the number of indicators (*k* = 1, 2, …, 8); ρ is the resolution ratio, which enlarges the difference among various coefficients.ρ=0.5 is generally used.

1. Calculating the gray–correlation degree. In obtaining the gray–relational degree, the following equation was used:

(9) $$X_{i}=\overset{k}{\underset{i=1}{\sum }}\zeta_{i}\left(k\right)\times W_{i}\;\left(k\right)$$

where $\zeta_{i}\;\left(k\right)$ is the gray–correlation coefficient of the indicator *Ci*; $W_{i}\;\left(k\right)$ is the object weight of the indicator *Ci*; *i* is the number of samples, and *i* = 0, 1, 2, …, and 15; *k* is the number of indicators, and *k* = 1, 2, …, 8.

### 2.3 Statistical analyses

Each sample was made in three parallel. Microsoft Excel 2010 and SPSS 19 statistical analysis software were used for plotting and data analysis. The experimental results were analyzed by variance analysis and compared by using the Student–Newman–Keuls test. Bivariate correlation and Pearson correlation coefficients were used to analyze the correlation analysis. *p* ≤ 0.05 was considered significant, and *p* ≤ 0.01 was considered extremely significant.

## 3 Results

### 3.1 Effect of acetic acid on the storage quality of fresh–cut water chestnuts

**Figure 1:**
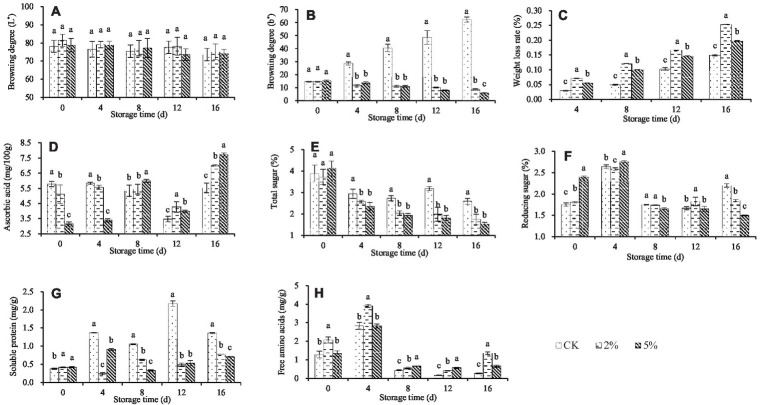
Effect of acetic acid on the storage quality of fresh–cut water chestnuts. Each value represents the mean of three replicates ± standard deviation (SD). Columns with different letters are significantly different at the same storage time (*p* < 0.05). **(A)** Browning degree (L*); **(B)** Browning degree (b*); **(C)** Weight loss rate; **(D)** Ascorbic acid; **(E)** Total sugar; **(F)** Reducing sugar; **(G)** Soluble protein; **(H)** Free amino acids.

The storage quality of fresh–cut water chestnuts exposed to different concentrations of acetic acid and stored for different time varied (Figure 1).

#### 3.1.1 Color determination

Color is an important factor affecting the appearance quality of fruits and vegetables, and is an indicator for evaluating the quality of fresh–cut fruits and vegetables (41). L^*^ represents the brightness of an object. The value of L * is between 0 and 100, and the lower the L * value, the more severe the browning. Positive b^*^ represents the yellow color of an object, the higher the b^*^ value, the more serious the yellowing of fruits and vegetables (41). During the storage process, the color changes of fresh–cut water chestnuts treated with 0, 2 and 5% acetic acid are shown in Figures 1A,B. As the storage time increased, the L * value usually decreased while the b * value increased. Different from the L * value, there was a significant difference in the b * value between CK treatment and acetic acid treatment when fresh–cut water chestnuts were stored for the same time. It was indicated that acetic acid can inhibit the browning and yellowing of fresh–cut water chestnuts during storage. In addition, there was almost no significant difference in the effects of 2 and 5% acetic acid on the L * and b * values of fresh–cut water chestnuts.

#### 3.1.2 Weight loss rate

During storage, the weight of fruits and vegetables gradually decreases due to respiration and transpiration. Considering the mechanical damage during the processing, the quality deterioration of fresh–cut water chestnuts has intensified, resulting in a short shelf life and significant economic losses. The weight loss rate of fresh–cut water chestnuts treated with different concentrations of acetic acid is shown in Figure 1C, and increased with increasing storage time. At the same storage time, there was a significant difference in weight loss rates among different treatments, with 2% acetic acid, 5% acetic acid, and the control in descending order. It was indicated that acetic acid treatment caused severe weight loss in fresh–cut water chestnuts.

#### 3.1.3 Nutritional ingredients

The respiration and transpiration intensity of fresh–cut water chestnuts changed continuously during storage and caused tissue internal nutrient unceasing transformation, which directly affected the content of soluble solids in fresh–cut water chestnuts (42). During the storage period of 0 to 8 days, the ascorbic acid content of fresh–cut water chestnuts treated with 0 and 2% acetic acid slightly changed, but the ascorbic acid content of 5% acetic acid treatment showed an upward trend (Figure 1D). During the storage period of 8 to 16 days, the ascorbic acid content of different treatments showed a trend of first decreasing and then increasing, while the ascorbic acid concentration of acetic acid treatment was significantly higher than that of the control. That is to say, if stored for more than 4 days, acetic acid treatment would lead to a higher content of ascorbic acid in fresh–cut water chestnuts. Meanwhile, 5% acetic acid was higher than 2% acetic acid.

During the storage process of fresh–cut water chestnuts, the total sugar of CK decreased but remained relatively constant, while the total sugar of acetic acid treatment showed a continuous downward trend (Figure 1E). When stored for the same time, compared with the control, acetic acid resulted in a lower total sugar content, but there was no significant difference between 2 and 5% acetic acid. In terms of reducing sugars, all treatments showed a trend of first rising and then falling (Figure 1F), and the maximum value occurred when the storage time was 4 days. When the storage time exceeded 4 days, the reducing sugar content in acetic acid treatment was basically lower than that in the control. For soluble protein, all treatments showed irregular changes (Figure 1G). Basically, the content had increased to varying degrees. When the storage time was the same, the reducing sugar content in acetic acid treatment was lower than that in the control. For free amino acid, all treatments showed a trend of first increasing and then decreasing (Figure 1H), and the maximum value occurred when the storage time was 4 days. When stored for the same time, the free amino acid content in acetic acid treatment was higher than that in the control. Meanwhile, the significant differences in soluble protein and free amino acid were usually observed between the treatments of 2 and 5% acetic acid.

#### 3.1.4 Gray–correlation analyses

For fresh–cut water chestnuts exposed to 0, 2 and 5% acetic acid, the different changes in the color, weight loss rate, and content of nutritional components occurred during the storage (Figure 1). Taking into account the changing trends of various indicators, it was difficult to directly determine which treatment method to choose to extend the shelf life of fresh–cut water chestnuts and ensure their storage quality. Thus, it was necessary to conduct a comprehensive evaluation of fresh–cut water chestnuts treated with acetic acid.

#### 3.1.5 Weight value of impact factors

The coefficient of variation method is an objective assignment method that calculates weights by utilizing the information contained in various indicators. It is widely used in comprehensive evaluation of multiple indicators (43). Using the coefficient of variation method to calculate the average, standard deviation, coefficient of variation, and weight of various indicators in fresh–cut water chestnuts treated with 0, 2 and 5% acetic acid and stored for different time, the results are listed in Table 1. The weights of L^*^, b^*^, weight loss rate, ascorbic acid, total sugar, reducing sugar, soluble protein, and free amino acid accounted for 1.3, 21.0, 19.9, 6.4, 8.0, 5.2, 16.5, and 21.8%, respectively. It was indicated that different indicators contributed differently to the quality of fresh–cut water chestnuts.

**Table 1:** Weights of various indicators used in the comprehensive evaluation of fresh–cut water chestnuts.

Treatment	L^*^	b^*^	Weight loss rate/%	Ascorbic acid/(mg/100 g)	Total sugar/%	Reducing sugar/%	Soluble protein/(mg/g)	Free amino acid/(mg/g)
Mean value	76.893	20.204	0.096	5.172	2.606	1.984	0.785	1.289
Standard deviation	3.768	16.486	0.074	1.290	0.814	0.401	0.503	1.092
*Vi*	0.049	0.816	0.772	0.249	0.312	0.202	0.641	0.847
*Wi*	0.013	0.210	0.199	0.064	0.080	0.052	0.165	0.218

#### 3.1.6 Normalization of raw data

Given the non–uniform dimension of the raw data shown in Figure 1, these data should be made dimensionless before comprehensive evaluation (39, 40). Selecting the preservative agent with excellent quality properties for fresh–cut water chestnuts was desirable to meet quality targets. Therefore, in the ideal process selected as the reference sequence (X_0_), the L^*^, ascorbic acid, total sugar, reducing sugar, soluble protein, and free amino acid were the highest values of the corresponding indicators in the comparison sequence, and the b^*^ was the lowest value of the corresponding indicators in the comparison sequence. With regard to the weight loss rate, the lowest value in the comparison sequence was 0, and the following calculation cannot be performed using the value of 0. Thus, the weight loss rate in the ideal process was the minimum value of the corresponding indicators in the comparison sequence, excluding 0. All raw data were normalized using Equation 4. The standardized data of evaluation indices are listed in Table 2.

**Table 2:** Standardized data of evaluation indices.

Treatment	Storage time	L^*^	b^*^	Weight loss rate	Ascorbic acid	Total sugar	Reducing sugar	Soluble protein	Free amino acid
Ideal		1.000	1.000	1.000	1.000	1.000	1.000	1.000	1.000
CK	0	0.960	2.467	0.000	0.746	0.941	0.635	0.175	0.329
	4	0.940	4.895	1.000	0.752	0.710	0.956	0.631	0.724
	8	0.927	6.947	1.694	0.690	0.661	0.635	0.485	0.114
	12	0.953	8.337	3.530	0.449	0.770	0.606	1.000	0.044
	16	0.903	10.703	5.149	0.713	0.627	0.797	0.627	0.068
2%	0	1.000	2.472	0.000	0.660	0.902	0.655	0.192	0.531
	4	0.971	2.009	2.444	0.718	0.622	0.937	0.111	1.000
	8	0.947	1.884	4.155	0.703	0.493	0.630	0.286	0.143
	12	0.960	1.689	5.705	0.553	0.482	0.662	0.225	0.106
	16	0.922	1.471	8.713	0.907	0.422	0.669	0.358	0.347
5%	0	0.967	2.596	0.000	0.406	1.000	0.868	0.197	0.343
	4	0.968	2.336	1.913	0.439	0.571	1.000	0.417	0.724
	8	0.948	1.854	3.457	0.773	0.467	0.602	0.154	0.171
	12	0.903	1.353	5.055	0.516	0.438	0.599	0.246	0.147
	16	0.910	1.000	6.801	1.000	0.367	0.544	0.326	0.169

#### 3.1.7 Absolute difference between the comparison and reference sequence

The absolute difference between the comparison and reference sequence was calculated using Equation 5, and the results are shown in Table 3. In accordance with Equations 6, 7, the minimum absolute difference was 0.000, and the maximum absolute difference was 9.703.

**Table 3:** Absolute value between the reference sequence and comparative sequence.

Treatment	Storage time	L^*^	b^*^	Weight loss rate	Ascorbic acid	Total sugar	Reducing sugar	Soluble protein	Free amino acid
CK	0	0.040	1.467	1.000	0.254	0.059	0.365	0.825	0.671
	4	0.060	3.895	0.000	0.248	0.290	0.044	0.369	0.276
	8	0.073	5.947	0.694	0.310	0.339	0.365	0.515	0.886
	12	0.047	7.337	2.530	0.551	0.230	0.394	0.000	0.956
	16	0.097	9.703	4.149	0.287	0.373	0.203	0.373	0.932
2%	0	0.000	1.472	1.000	0.340	0.098	0.345	0.808	0.469
	4	0.029	1.009	1.444	0.282	0.378	0.063	0.889	0.000
	8	0.053	0.884	3.155	0.297	0.507	0.370	0.714	0.857
	12	0.040	0.689	4.705	0.447	0.518	0.338	0.775	0.894
	16	0.078	0.471	7.713	0.093	0.578	0.331	0.642	0.653
5%	0	0.033	1.596	1.000	0.594	0.000	0.132	0.803	0.657
	4	0.032	1.336	0.913	0.561	0.429	0.000	0.583	0.276
	8	0.052	0.854	2.457	0.227	0.533	0.398	0.846	0.829
	12	0.097	0.353	4.055	0.484	0.562	0.401	0.754	0.853
	16	0.090	0.000	5.801	0.000	0.633	0.456	0.674	0.831

#### 3.1.8 Gray–correlation coefficient

Equation 8 was used to count the correlation coefficient between the treatments (CK, 2 and 5% acetic acid) and the quality indicators of fresh–cut water chestnuts. Then, the calculated correlation coefficients are given in Table 4.

**Table 4:** Gray–relational coefficient and gray–correlation degree between the reference sequence and comparative sequence.

Treatment	Storage time	L^*^	b^*^	Weight loss rate	Ascorbic acid	Total sugar	Reducing sugar	Soluble protein	Free amino acid	Gray–correlation degree
CK	0	0.992	0.768	0.829	0.950	0.988	0.930	0.855	0.878	0.859
	4	0.988	0.555	1.000	0.951	0.944	0.991	0.929	0.946	0.875
	8	0.985	0.449	0.875	0.940	0.935	0.930	0.904	0.846	0.797
	12	0.990	0.398	0.657	0.898	0.955	0.925	1.000	0.835	0.756
	16	0.980	0.333	0.539	0.944	0.929	0.960	0.929	0.839	0.710
2%	0	1.000	0.767	0.829	0.934	0.980	0.934	0.857	0.912	0.865
	4	0.994	0.828	0.771	0.945	0.928	0.987	0.845	1.000	0.883
	8	0.989	0.846	0.606	0.942	0.905	0.929	0.872	0.850	0.820
	12	0.992	0.876	0.508	0.916	0.903	0.935	0.862	0.844	0.803
	16	0.984	0.912	0.386	0.981	0.893	0.936	0.883	0.881	0.801
5%	0	0.993	0.753	0.829	0.891	1.000	0.974	0.858	0.881	0.856
	4	0.993	0.784	0.842	0.896	0.919	1.000	0.893	0.946	0.881
	8	0.989	0.850	0.664	0.955	0.901	0.924	0.851	0.854	0.831
	12	0.980	0.932	0.545	0.909	0.896	0.924	0.865	0.850	0.822
	16	0.982	1.000	0.455	1.000	0.885	0.914	0.878	0.854	0.826

#### 3.1.9 Gray–correlation degree

**Figure 2:**
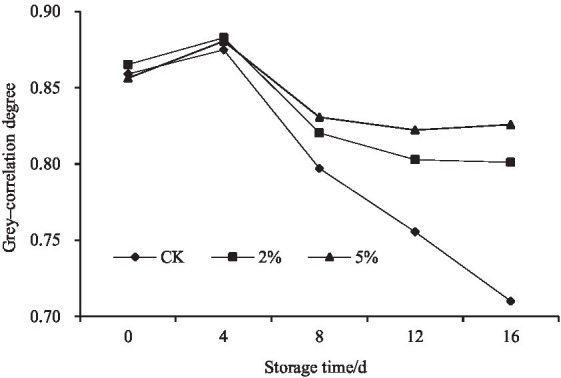
Effect of acetic acid on the comprehensive score of fresh–cut water chestnuts.

Gray–correlation degree was calculated using Equation 9, and shown in Table 4 and Figure 2. It was found that the gray–correlation degrees of CK, 2% or 5% acetic acid decreased with the increase of storage time. When the storage time was the same, the comprehensive scores of the acetic acid treatments were higher than CK. Therefore, the fresh–cut water chestnuts could be preserved by acetic acid. With regard to the concentration of acetic acid, the gray–correlation degree of 5% acetic acid was higher than that of 2% at the same storage time. Based on the gray–correlation degree, it could be directly concluded that when the storage time was more than 4 days, acetic acid could be used to improve the storage quality, and the freshness–keeping effect of 5% acetic acid was better than 2%. And it was not necessary for fresh–cut water chestnuts stored for 4 days to be treated with acetic acid.

### 3.2 Correlation analysis between measurement indicators and gray–correlation degree in fresh–cut water chestnuts

**Figure 3:**
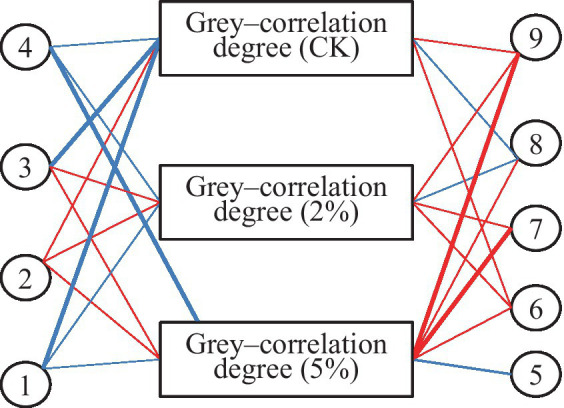
Network correlation between parameter and gray-correlation degree. 1: Storage time; 2: L^*^; 3: b^*^; 4: Weight loss rate; 5: Ascorbic acid; 6: Total sugar; 7: Reducing sugar; 8: Soluble protein; 9: Free amino acid. Red line: Positive correlation; Blue line: Negative correlation; Thick line: Correlation coefficient ≥ 0.9 or ≤ −0.9; Thin line: Correlation coefficient between 0.5 and 0.9 or between −0.9 and − 0.5.

The correlation analysis between measurement indicators and gray–correlation degree in fresh–cut water chestnut is shown in Table 5 and Figure 3. It was shown that a significant negative correlation was between gray–correlation degree and storage time in treatments of 0 and 2% acetic acid. The significant negative correlation was also between gray–correlation degree and b^*^, and the weight loss in CK. The significant positive correlation was between gray–correlation degree and free amino acids content in 2% acetic acid treatment. The extreme significant positive correlation was between gray–correlation degree and reducing sugar, and free amino acids in 5% acetic acid treatment. However, the correlation between L^*^, ascorbic acid, total sugar, soluble protein, and gray–correlation degree was not significant in the treatments of 0, 2 and 5% acetic acid. Acetic acid treatment reduced the correlation levels between storage time, b–value, weight loss rate, and correlation degree, but increased the levels between reducing sugar, free amino acids, and correlation degree.

**Table 5:** Correlation analysis between measurement indicators and gray-correlation degree in fresh–cut water chestnuts.

Treatment	Storage time	L^*^	b^*^	Weight loss rate	Ascorbic acid	Total sugar	Reducing sugar	Soluble protein	Free amino acid
CK	−0.953^*^	0.627	−0.936^*^	−0.951^*^	0.471	0.547	0.268	−0.516	0.837
2%	−0.878^*^	0.747	0.779	−0.834	−0.138	0.748	0.701	−0.846	0.879^*^
5%	−0.755	0.844	0.802	−0.734	−0.624	0.521	0.979^**^	0.534	0.972^**^

*Indicates significant correlation (*p* < 0.05); **indicates extremely significant correlation (*p* < 0.01).

## 4 Discussion

### 4.1 Storage quality of fresh–cut water chestnuts

After fresh–cut processing, water chestnuts suffer serious mechanical damage. Due to their own stress resistance and a series of physiological reactions, fresh–cut water chestnuts are prone to discoloration during storage, which affects the shelf life and storage quality. The present results indicated that the discoloration of fresh–cut water chestnuts appeared gradually, and the color worsened during storage (Figures 1A,B). Meanwhile, the weight loss rate increased (Figure 1C) and the content of nutritional ingredients changed (Figures 1D,H).

Acetic acid can inhibit enzyme activity, intervene in nutrient transport, disrupt membrane systems, disrupt metabolism, and effectively inhibit the decay of post–harvest fruit caused by microorganisms (16). Currently, it has been tested for anti–corrosion and preservation of various foods (44). In the present study, the L^*^ values representing the browning degree indicated that fresh–cut water chestnuts exhibited browning in both with and without acetic acid treatment (Figure 1A). Despite no significant difference, acetic acid treatment basically inhibited a little browning. It is consistent with the report in which the browning of fresh–cut water chestnuts soaked in white vinegar solution could be delayed (45). There might be two reasons for this based on the acidic pH value of acetic acid. Firstly, the solution induced microbial dehydration and death by adjusting osmotic pressure. Secondly, it also could inhibit enzymatic browning by reducing enzyme activity. To clarify the exact cause, the next step should focus on studying the effects of acetic acid on enzymatic browning and microorganisms during the storage process of fresh–cut water chestnuts.

During storage, fresh–cut water chestnuts not only exhibit common browning in fresh–cut products, but also gradually appear yellowing phenomenon (41). The significant differences in b^*^ values among treatments indicated that the yellowing of fresh–cut water chestnuts could be inhibited by acetic acid, as well as ultraviolet irradiation (11), ethephon (41), and ethanol (46). It was reported that the yellowing phenomenon of fresh–cut water chestnuts was related to the oxidation of flavonoids in water chestnuts (47). And the metabolites produced by microorganisms may convert flavonoid glycosides into free flavonoids, also accelerating the yellowing phenomenon (46). Rice bran extracts effectively delayed the yellowing of fresh–cut water chestnuts during storage, with a significant inhibitory effect on the accumulation of flavonoids and flavonoid metabolism (9). The reason why acetic acid inhibits the yellowing of fresh–cut water chestnuts may be due to its dual inhibitory effects on flavonoids and microorganisms. To clarify the exact reason, the next step should focus on studying the effects of acetic acid on flavonoids, flavonoid related enzymes, and microorganisms in fresh–cut water chestnuts during storage.

Fresh–cutting processing not only affects the color of water chestnuts, but also affects their other storage quality. The weight loss of fruits and vegetables is caused by transpiration during storage and transportation, which leads to a decrease in water and nutrients in the tissues. The weight loss of fresh–cut water chestnuts easily occurred after peeling and seriously affected its sensory and edible quality (42). Appropriate intensity of pulsed light treatment could reduce damage to tissue cells, thereby reducing water loss and nutrient consumption, and improving the quality of fresh–cut water chestnuts (48). Unlike pulsed light, but similar to UV and ethanol (11), acetic acid treatment could not inhibit water loss and nutrient consumption in fresh–cut water chestnuts (Figure 1C). A higher weight loss rate indicated significant loss of water and nutrients, as well as stronger respiration and transpiration. Unlike acetaldehyde, acetic acid treatment may promote the activity of enzymes related to plant cell wall depolymerization (e.g., pectin methyl esterase, polygalacturonase, and β–galactosidase), thereby disrupting the integrity of fresh–cut water chestnut tissue cells, which is not conducive to maintaining the water–holding capacity of tissue cells (42).

Soluble solids continuously decreased during the storage process of fresh–cut water chestnuts, showing a trend of first fast and then slow (45). Perhaps the tissue cells provide energy for their respiration by consuming their own nutrients, leading to a gradual decrease in soluble solids content in the tissue. According to Figure 1C, the effect of acetic acid on the weight loss of fresh–cut water chestnuts showed that acetic acid enhanced the respiratory of fresh–cut water chestnuts, inducing significant loss of water and nutrients. Contrary to free amino acids, when stored for the same time, the total sugar and soluble protein content in CK was higher than those in acetic acid (Figures 1E,G,H). For ascorbic acid, when stored for 4 days, CK was higher than acetic acid treatment, and when stored for more than 4 days, acetic acid treatment was higher than CK (Figure 1D). Compared with other indicators, there was no significant difference in reducing sugar content between the control and acetic acid treatment with the same storage time (Figure 1F). The results also indicated that the respiration of fresh–cut water chestnuts was affected by acetic acid, so the nutritional content of CK and the acetic acid treatments was different. Based on the increase in weight loss rate and the decrease in total sugar and soluble protein content, it was inferred that acetic acid promoted the respiration of fresh–cut water chestnuts, and increased the content of VC and free amino acids.

### 4.2 Comprehensive evaluation

Undoubtedly, preservation treatment should not only maintain the good sensory quality of fresh–cut products, but also have good nutritional quality. 0, 2 and 5% acetic acid had different effects on the color, weight loss rate, and nutritional content of fresh–cut water chestnuts. Considering the changing trends of various indicators, it was difficult to directly determine which treatment method to choose to prolong the shelf life of fresh–cut water chestnuts and ensure their storage quality. Therefore, it is necessary to conduct a comprehensive evaluation of the preservation effect of fresh–cut water chestnuts. There are currently some comprehensive evaluation methods that can be applied in multiple industries. Principal component analysis was applied in the quality evaluation of fresh–cut cauliflower in different packaging methods (49), quality evaluation of fresh–cut lettuce (50), and the effect evaluation of composite coatings in preserving the quality of Wax apples (51). Entropy method and analytic hierarchy process was used to optimize the fresh–cut processing method of Gastrodiae Rhizoma (52). Fuzzy mathematics was used to evaluate the effect of natural preservatives on chilled pork preservation (53) and analyze the suitability of fresh–cut vegetable processing for South China ecotypic cucumber (54). These methods each have their own advantages and disadvantages.

Gray–correlation analysis is an evaluation method of determining the primary and secondary factors as well as the degree of their correlation in accordance with the gray system (27). It is reported (55) that the advantage of this method is: firstly, it can be directly calculated from raw data, is easy to master, has a small computational workload, and the results are objective and reliable; secondly, low data requirements, limited data or lack of clear distribution patterns can also be used for calculations. And the disadvantage of this method is: firstly, different values of comparison sequences, resolution coefficients, etc. can affect the evaluation unit, resulting in inconsistent results; secondly, there can be both positive and negative correlations between things, and using a correlation model when there is a negative correlation can lead to incorrect conclusions; thirdly, it is not suitable for situations where the evaluation indicator information is duplicated, so the selection of indicators has a significant impact on the evaluation results. Given the simple calculation and reasonable evaluation, gray–correlation analysis had been used to comprehensively evaluate the effects of processing methods on turnip chips (40), Chinese yam chips (30), *Hypsizygus marmoreus* (31), black sesame seeds (56), South China ecotypic cucumber (57), and fresh–peach (39). The application of gray correlation analysis in the preservation of fresh–cut water chestnuts has not been reported yet.

In order to provide scientific basis for acetic acid preservation, the color, weight loss rate, and content of ascorbic acid, total sugar, reducing sugar, soluble protein, and free amino acid in fresh–cut water chestnuts treated with acetic acid were measured. In addition, the experimental data were analyzed and compared (Figure 1). However, the information was too cumbersome and complex, making it difficult to determine which of the 0, 2 and 5% acetic acid treatments works well. Consequently, the importance of different concentrations of acetic acid solutions affecting the quality of fresh–cut water chestnuts was classified by gray–correlation analysis based on the variation–coefficient method. Firstly, the weights of the quality indicators of fresh–cut water chestnuts were calculated by using the variation–coefficient method (Table 1). Secondly, the gray–correlation degree of each factor was calculated through a series of data processing using gray correlation analysis (Tables 2–4). Then, the results of gray–correlation analysis were taken as the reference for the final comprehensive evaluation (Table 4; Figure 2).The gray–correlation degree of CK, 2% or 5% acetic acid, typically decreased with increasing storage time. Finally, acetic acid could be used to preserve fresh–cut water chestnuts stored for more than 4 days, and the preservation effect of 5% acetic acid was better than that of 2%. Our findings may provide information and a comprehensive evaluation approach to the preservation of fresh–cut water chestnuts and other fresh–cut fruits and vegetables.

## 5 Conclusion

Treatments with pure water and 2 and 5% acetic acid had varying degrees of impact on the color, weight loss rate, and nutritional ingredients of fresh–cut water chestnuts. In addition, the influences of various treatments on the quality indicators of fresh–cut water chestnuts could be compared and evaluated comprehensively using gray–correlation analysis by analyzing the color, weight loss rate, and content of ascorbic acid, total sugar, reducing sugar, soluble protein, and free amino acid. It was found that fresh–cut water chestnuts stored for 4 days did not require acetic acid treatment, but when the storage time was more than 4 days, acetic acid could be used to preserve, and the preservation effect of 5% acetic acid was better than that of 2%. These findings indicated that acetic acid, as a simple, safe, and economically effective preservative, was suitable for the preservation of fresh–cut water chestnuts. However, the effects of these treatments on other indicators (e.g., phenols, flavonoids, flavonoid related enzymes, and microorganisms) in fresh–cut water chestnuts have not been studied yet. Therefore, further research is needed to assess the fresh–keeping effect of fresh–cut water chestnuts by examining other components, such as total phenols, flavonoids, and microorganisms, to provide an idea for the study of preservatives. In summary, a comprehensive analysis method was established by analyzing multiple indicators to determine the preservation effect of acetic acid in fresh–cut water chestnuts.

## Data availability statement

The raw data supporting the conclusions of this article will be made available by the authors, without undue reservation.

## Author contributions

YZ: Writing – review & editing, Writing – original draft, Methodology, Investigation, Formal analysis, Data curation. HL: Writing – original draft, Formal analysis, Data curation. JL: Writing – original draft, Software, Data curation. QW: Writing – review & editing, Software, Formal analysis, Data curation. JS: Writing – review & editing, Resources. DW: Writing – review & editing, Supervision, Conceptualization.

## References

[1] Gao M Jiang W Wei S Lin Z Cai B Yang L. High–efficiency propagation of Chinese water chestnut [*Eleocharis dulcis* (Burm.F.) Trin. Ex Hensch] using a temporary immersion bioreactor system. Plant Cell Tiss Org. (2015) 121:761–72. doi: 10.1007/s11240-015-0732-4

[2] Hussain N Shukla SS Dubey AD Gautam S Tripathi J. Control of post–harvest storage losses in water chestnut (*Trapa bispinosa* Roxburg) fruits by natural functional herbal coating and gamma radiation processing. J Food Sci Technol. (2022) 59:2842–54. doi: 10.1007/s13197-021-05307-x, PMID: 35734140 PMC9206974

[3] Peng L Jiang Y. Effects of heat treatment on the quality of fresh–cut Chinese water chestnut. Int J Food Sci Tech. (2004) 39:143–8. doi: 10.1046/j.0950-5423.2003.00767.x

[4] You Y Jiang Y Sun J Liu H Song L Duan X. Effects of short–term anoxia treatment on browning of fresh–cut Chinese water chestnut in relation to antioxidant activity. Food Chem. (2012) 132:1191–6. doi: 10.1016/j.foodchem.2011.11.073, PMID: 29243600

[5] Kong M Murtaza A Hu X Iqbal A Zhu L Ali S. Effect of high-pressure carbon dioxide treatment on browning inhibition of fresh-cut Chinese water chestnut (*Eleocharis tuberosa*): based on the comparison of damaged tissue and non-damaged tissue. Postharvest Biol Tec. (2021) 179:111557. doi: 10.1016/j.postharvbio.2021.111557

[6] Peng L Jiang Y. Exogenous salicylic acid inhibits browning of fresh–cut Chinese water chestnut. Food Chem. (2006) 94:535–40. doi: 10.1016/j.foodchem.2004.11.047

[7] Peng L Yang S Li Q Jiang Y Joyce YD. Hydrogen peroxide treatments inhibit the browning of fresh–cut Chinese water chestnut. Postharvest Biol Tec. (2008) 47:260–6. doi: 10.1016/j.postharvbio.2007.07.002

[8] Pen LT Jiang YM. Effects of chitosan coating on shelf life and quality of fresh–cut Chinese water chestnut. Lebensm-Wiss Technol. (2003) 36:359–64. doi: 10.1016/S0023-6438(03)00024-0

[9] Song M Sun Y Liu Y Nong J Liu Y He M. Effect of rice bran extracts on yellowing of fresh-cut Chinese water chestnut. Guangdong Agricultural Sciences. (2024) 1:1–9. doi: 10.16768/j.issn.1004-874X.2024.01.001

[10] Chen Z. Study and application of eugenol treatment on fresh-peeled Chinese water chestnut preservation. Wuhan, China: Wuhan Polytechnic University (2023).

[11] Niu L Yang Y Yi Y Hou W Wang H Min T. The effect of ultraviolet irradiation and ethanol treatment on browning of fresh-cutting water chestnuts. Food Sci Technol. (2019) 11:41–6. doi: 10.13684/j.cnki.spkj.2019.11.008

[12] Teng Y Kong M Chen X Hu W Xu X Pan S. Research progress on the mechanism and control of discoloration in fresh–cut Chinese water chestnut. Sci Technol Food Ind. (2019) 2:321–5. doi: 10.13386/j.issn1002-0306.2019.02.056

[13] Cortesia C Vilchèze C Bernut A Contreras W Gómez K Waard J. Acetic acid, the active component of vinegar, is an effective Tuberculocidal disinfectant. MBio. (2014) 5:e00013–4. doi: 10.1128/mBio.00013-14, PMID: 24570366 PMC3940030

[14] Luo F Guan Y Ong W Du Z Ho G Li F. Enhancement of pulsed laser ablation in environmentally friendly liquid. Opt Express. (2014) 22:23875–82. doi: 10.1364/OE.22.023875, PMID: 25321965

[15] Diba F Alam F Talukder AA. Screening of acetic acid producing microorganisms from decomposed fruits for vinegar production. Adv Microbiol. (2015) 5:291–7. doi: 10.4236/aim.2015.55028

[16] Venditti T Dorea A Molinua MG Agabbiob M D’Hallewina G. Combined effect of curing followed by acetic acid vapour treatments improves postharvest control of *Penicillium digitatum* on mandarins. Postharvest Biol Technol. (2009) 54:111–4. doi: 10.1016/j.postharvbio.2009.06.002

[17] Krusong W Teerarak M Laosinwattana C. Liquid and vapor–phase vinegar reduces *Klebsiella pneumonia* on fresh coriander. Food Control. (2015) 50:502–8. doi: 10.1016/j.foodcont.2014.09.051

[18] Sholberg PL Shephard T Randall P Moyls L. Use of measured concentrations of acetic acid vapour to control postharvest decay in d’Anjou pears. Postharvest Biol Technol. (2004) 32:89–98. doi: 10.1016/j.postharvbio.2003.09.014

[19] Si W Yang F Wang J. Fresh corn preservation technology at normal temperature. Food Ferment Ind. (2019) 6:1–8. doi: 10.13995/j.cnki.11-1802/ts.018973

[20] Tzortzakis NG Tzanakaki K Economakis CD. Effect of origanum oil and vinegar on the maintenance of postharvest quality of tomato. Food Nutr Sci. (2011) 2:974–82. doi: 10.4236/fns.2011.29132

[21] Peng Y Sun JJ Shi JY Zhang X Wang Q. Application of acetic acid to replace sulfur dioxide on Kyoho grape decay prevention. Food Ferment Ind. (2018) 9:188–95. doi: 10.13995/j.cnki.11-1802/ts.016236

[22] Zhao GH Chen ZL. Study on fresh–cut Chinese water chestnut. Food Res Dev. (2012) 1:197–200.

[23] Pan YG Shi JY Chen WX. Isolation and identification of microbes from yellowing tissues of fresh–cut Chinese water chestnut. J Southwest Univ. (2008) 3:66–9. doi: 10.13718/j.cnki.xdzk.2008.03.018

[24] Zhou P Yao YJ Ai YF Liu AM Xu ZL Xie JC. Grey correlation analysis of factors influencing maldistribution in feeding device of copper flash smelting. J Cent South Univ. (2012) 19:1938–45. doi: 10.1007/s11771-012-1229-5

[25] Weng H Jiang J Feng M Yao M Chen C Lian G. Multi–objective optimizations of the Q355C steel gas metal arc welding process based on the grey correlation analysis. Int J Adv Manuf Technol. (2022) 121:3567–82. doi: 10.1007/s00170-022-09547-9

[26] Xu G Yang Z. Multiobjective optimization of process parameters for plastic injection molding via soft computing and grey correlation analysis. Int J Adv Manuf Technol. (2015) 78:525–36. doi: 10.1007/s00170-014-6643-4

[27] Firat MEO. Experimental investigation on the thermal characteristics and grey correlation analysis of frost penetration depths for different subgrade soils. Environ Earth Sci. (2021) 80:394. doi: 10.1007/s12665-021-09698-0

[28] Liu J Ren J Jiang J Cheng Y Qi D. Optimization of grey correlation analysis model of chemical process safety evaluation index system. Saf Environ Eng. (2024) 2:70. doi: 10.13578/j.cnki.issn.1671-1556.20221090

[29] Xu J Yao X Huang M Huang Y Pan L Zhang Z. Quality evaluation of fresh *Rehmannia glutinosa* based on chemical pattern recognition and grey correlation method. Mod Chin Med. (2024) 1:18–28. doi: 10.13313/j.issn.1673-4890.20230307001

[30] Gao Q Chen JN Zhang JC Liu CJ Liu CQ Xue YL. Comprehensive evaluation of the effects of different drying methods on the quality of Chinese yam chips based on gray relational analysis. Sci Technol Food Ind. (2018) 16:6–12. doi: 10.13386/j.issn1002-0306.2018.16.002

[31] Lai P Tang B Li Y Wu L Weng M Chen J. Grey correlation analysis for physical and nutritional quality of hypsizygus marmoreus from different drying methods. J Nucl Agric Sci. (2021) 35:2118–26. doi: 10.11869/j.issn.100-8551.2021.09.2118

[32] Ye R Sun L Wang Y Xu D Ji H He J. Grey relational analysis between chromatism and some representative qualitative traits of Litopenaers Vannamei in refrigeration. J Chin Inst Food Sci Technol. (2018) 3:205–10. doi: 10.16429/j.1009-7848.2018.03.027

[33] Shen Y Gao M Yang L Zhao X Chen X Wang Z. Suitability analysis of fresh-cut vegetable processing for twenty main green capsicum cultivars in China. Trans Chin Soc Agric Eng. (2016) 32:359–68.

[34] Weng X Xin G Li YX. Study on determination conditions of total sugar from potato starch by anthrone colorimetry. Food Res Dev. (2013) 17:86–8. doi: 10.3969/j.issn.1005-6521.2013.17.023

[35] Cao J Jiang W Zhao Y. Experiment guidance of postharvest physiology and biochemistry of fruits and vegetables, 1st Edn. Beijing, China: China Light Industry Press (2007), pp. 41–70.

[36] Li Q Meng XX Liu YB Pang LF. Risk assessment of floor water inrush using entropy weight and variation coefficient model. Geotech Geol Eng. (2019) 37:1493–501. doi: 10.1007/s10706-018-0702-9

[37] Li J Hu Y Wang X Diao M Diao M. Study on the operation safety evaluation system of ship lock combined with variation coefficient method and matter-element extension method In: Li Y Hu Y Rigo P Lefler FE Zhao G, editors. Proceedings of PIANC smart Rivers 2022. PIANC 2022. Lecture notes in civil engineering, vol. 264. Singapore: Springer (2022)

[38] Wang H Gao Y Han Y. Determining the main controlling factors of nitrogen diffusion fluxes at sediment–water interface by grey correlation analysis. Water Resour Manag. (2022) 36:4951–64. doi: 10.1007/s11269-022-03285-z

[39] Zheng X Yan X Zheng X Liu X Tan M Cao N. Comprehensive quality evaluation of fresh peach fruits based on grey correlation analysis. China Fruit Vegetable. (2023) 10:36–41+52. doi: 10.19590/j.cnki.1008-1038.2023.10.008

[40] Gao Q Li J Han H Liu Z Zhang J Liu C. Effects of different drying methods on turnip chips as evaluated based on grey relational analysis. Food Sci. (2019) 40:95–101. doi: 10.7506/spkx1002-6630-20171115-197

[41] Yuan RM Hu MW Min T Wang HX Yi Y Ai YW. Ethephon maintains the storage quality of fresh–cut *Eleocharis tuberosa* by regulating phenolic and reactive oxygen species metabolism. J Food Saf Qual. (2023) 5:217–25. doi: 10.19812/j.cnki.jfsq11-5956/ts.2023.05.048

[42] Deng LL Ming J Tian WN Zeng KF. Effect of acetaldehyde fumigation on fresh–cut Chinese water chestnut quality during room temperature storage. Food Sci. (2010) 2:233–6.

[43] Bi W Wei L Shi T. Study on processing and eating quality of rice with variable temperature drying technology based on principal component analysis. Cereal Feed Ind. (2021) 4:1–4+9.

[44] Sun J. Study on decay control technique of post-harvest grape during logistics with acetic acid alternative to sulfur dioxide. Tai’an, China: Shandong Agricultural University (2016).

[45] Wang J. Research and application of fresh cut water chestnut fresh-keeping packaging and antibacterial polyethylene packaging film. Shanghai, China: Shanghai Ocean University (2019).

[46] Fan C Chen X Wang S Cai F Mei X Shi J. Effect of soaking in aqueous ethanol on the storage quality of fresh-cut water chestnut. Mod Food Sci Technol. (2018) 12:122–128+25. doi: 10.13982/j.mfst.1673-9078.2018.12.019

[47] Jang Y He F Zhong H Wang L Pan Y. Effect of ethanol treatment on fresh-cut Chinese water chestnut etiolation and the related enzymes. Sci Technol Food Ind. (2017) 10:326–30. doi: 10.13386/j.issn1002-0306.2017.10.054

[48] Peng G Wang L Tu Y Yan S Li J Wang Q. Study on preservation technology of fresh-cut water chestnut by compound reinforced pulse strong light. J Changjiang Vegetables. (2019) 4:72–6.

[49] Pao C Wang C Chen H Xie C. Principal component analysis of quality of fresh-cut cauliflower in different packaging methods. Cereals Oils. (2022) 12:97–103. doi: 10.1016/j.afres.2022.100125

[50] Wen X. Establishment of quality evaluation model of fresh-cut lettuce and application. Shenyang, China: Shenyang Agricultural University (2022).

[51] Yin Q Yang S Pan Y Xiao X Wang C Chen C. Use of principal component analysis for the evaluation of the effect of composite coatings in preserving the quality of wax apples (*Syzygium samarangenese*). Food Sci. (2022) 23:254–60. doi: 10.7506/spkx1002-6630-20211227-311

[52] Fu Y Zhou T Xu Q Yang C Zhang J Xiao C. Optimization of Gastrodiae Rhizoma fresh-cut processing method based on entropy method and analytic hierarchy process. Chin Tradit Herb Drug. (2024) 5:1493–501.

[53] Zhao W Ma Y Wang R Wang Y Liang H Zhao X. Suitability of different potato cultivars for fresh-cut processing. Food Sci Technol. (2021) 12:55–62. doi: 10.13684/j.cnki.spkj.2021.12.009

[54] Zhan Y Sun J Liu Y Xiao L Wang X. Evaluating the effect of natural preservatives on the preservation of chilled pork based on fuzzy mathematics. Meat Res. (2020) 11:72–7.

[55] Dong H Zhao Y Ma G Zhang D. Comparative study on quality evaluation methods of higher vocational education. J Changsha Univ. (2016) 2:136–9.

[56] Zhang Y Wang J Tan H Lu X Wang D Wei Q. Evaluation of steaming and drying of black sesame seeds for nine cycles using grey–correlation analysis based on variation–coefficient weight. Molecules. (2023) 28:5266. doi: 10.3390/molecules28135266, PMID: 37446935 PMC10343377

[57] Gao J Zhu Y Luo F Deng Q Liang G Liu X. Comparative analysis on the suitability of fresh-cut vegetable processing for South China ecotypic cucumber. Mod Food Sci Technol. (2018) 12:45–52. doi: 10.13982/j.mfst.1673-9078.2018.12.008

